# Immune profiling of experimental murine mastitis reveals conserved response to mammary pathogenic *Escherichia coli*, *Mycoplasma bovis*, and *Streptococcus uberis*

**DOI:** 10.3389/fmicb.2023.1126896

**Published:** 2023-03-24

**Authors:** Peleg Schneider, Hagit Salamon, Nathalie Weizmann, Einat Nissim-Eliraz, Inna Lysnyansky, Nahum Y. Shpigel

**Affiliations:** ^1^Department of Basic Sciences, The Koret School of Veterinary Medicine, The Hebrew University of Jerusalem, Rehovot, Israel; ^2^Mycoplasma Unit, Kimron Veterinary Institute, Beit Dagan, Israel

**Keywords:** mastitis, murine model, *Escherichia coli*, *Mycoplasma bovis*, *Streptococcus uberis*, immune profiling

## Abstract

Mastitis is one of the most prevalent and economically important diseases of dairy animals. The disease is caused by ascending bacterial infection through the teat canal. Among the most common mastitis-causing bacteria are Gram-negative coliforms, Gram-positive streptococci and staphylococci, and mycoplasma. The most prominent cellular hallmark of acute mammary infection is a massive recruitment of blood neutrophils into the tubular and alveolar milk spaces. The complex biological processes of leukocyte recruitment, activation, adhesion, and migration in the mammary gland remain largely elusive to date. While field research of mastitis in dairy animals contributed a lot to the development of mitigation, control, and even eradication programs, little progress was made toward understanding the molecular mechanisms underlying the pathogenesis of the disease. We report here experimental mastitis model systems in lactating mice challenged with field strains of common udder pathogens in dairy cows. We used these model systems to apply recently developed multiplex gene expression technology (Nanostring nCounter), which enabled us to study the expression of over 700 immune genes. Our analysis revealed a core of 100 genes that are similarly regulated and functionally or physically interacting in *E. coli*, *M. bovis*, and *Strep uberis* murine mastitis. Common significantly enriched gene sets include TNFɑ signaling *via* NFkB, Interferon gamma and alpha response, and IL6-JAK-STAT3 signaling. In addition, we show a significantly enriched expression of genes associated with neutrophil extracellular traps (NET) in glands challenged by the three pathogens. Ligand-receptor analysis revealed interactions shared by the three pathogens, including the interaction of the cytokines IL1β, IL1ɑ, and TNFɑ with their receptors, and proteins involved in immune cell recruitment such as complement C3 and ICAM1 (with CD11b), chemokines CCL3 and CCL4 (with CCR1), and CSF3 (with CSF3R). Taken together, our results show that mammary infection with *E. coli*, *M. bovis*, and *Strep uberis* culminated in the activation of a conserved core of immune genes and pathways including NET formation.

## Introduction

Mastitis, inflammation of the mammary gland, is an important disease in dairy animals and breast-feeding women. The disease is caused by ascending infection through the teat canal of pathogenic bacteria such as *Escherichia coli*, *Streptococcus uberis*, *Staphylococcus aureus*, and *Mycoplasma bovis* (Shpigel et al., 1998; Dyson et al., 2022; Gelgie et al., 2022; Kurban et al., 2022). Mastitis is a good example of an infectious disease caused by a dominant pathogen and heeding the concept of Koch’s postulates. In field cases, the etiological agent is usually isolated in pure cultures of milk samples and the disease can be recapitulated by experimental infection in dairy animals or laboratory mice. The first phase of the disease requires massive growth of invading bacteria and colonization of the milk spaces in the tubular system and alveoli. Invading bacteria require to grow in milk and withstand innate antimicrobial mechanisms active in the milk such as the complement system and essential nutrients chelators. Next, inflammation is activated by microbial-associated molecular patterns (MAMPs) released by the growing bacteria leading to massive recruitment of blood neutrophils into the alveolar and milk spaces (Rainard et al., 2022). MAMPs activate a plethora of innate receptors and signaling systems in mammary epithelial cells (MEC) and alveolar macrophages which are the first line of response to the invading pathogens. These cells are known to express and release numerous inflammatory mediators such as cytokines, interleukins, chemokines, and reactive oxygen and nitrogen species (RONS). These inflammatory mediators initiate the recruitment of blood neutrophils which requires endothelial and epithelial adherence and transmigration along the trajectory from the blood vessels, across the vasculature, parenchyma, and MEC. This process involves adhesion molecules, chemoattractants, and receptors expressed on immune cell surfaces, blood vessels, and stromal mammary tissue. The complex biological processes of leukocyte recruitment, activation, adhesion, and migration in the mammary gland remain largely elusive to date. While field research of mastitis in dairy animals contributed a lot to the development of mitigation, control, and even eradication (for some pathogens) programs, little progress was made toward understanding the molecular mechanisms underlying the pathogenesis of the disease. To this end, we have used and further developed experimental mastitis model systems in lactating mice and applied recently developed multiplex gene expression technology (Nanostring nCounter) that enabled us to study the expression of over 700 immune genes in the disease (Warren, 2018). We show here that a core of activated immune genes and pathways including the formation of neutrophil extracellular traps (NETs) are all conserved in *E. coli*, *M. bovis*, and *Strep. uberis* mastitis.

## Materials and methods

### Bacterial strains

*Escherichia coli* strain P4-NR O15:H21H54, phylogroup B1 was isolated from the milk of an experimentally infected dairy cow (Shpigel et al., 1997; Leimbach et al., 2017; Mintz et al., 2019) and prepared for challenge in lactating mice as previously reported by us (Salamon et al., 2020). Briefly, bacteria from frozen stock were grown on Luria Bertani (LB) agar plates at 37°C for 24 h after which one isolated bacteria colony was transferred to 10 mL of LB broth for overnight growth at 37°C with shaking at 110 rpm. Pre-cultured bacteria were diluted 1:100 with fresh LB broth and grew to log phase for 3 h at 37°C with shaking at 110 rpm. Next, culture was centrifuged for 10 min at 4,000 × rpm and bacteria were washed twice with sterile PBS. Bacteria were resuspended in sterile PBS and inoculum was divided to 50 μL doses in 0.3 mL insulin syringes, approximately ~10^4^ CFUs and kept on ice until the IMM challenge. Challenge was quantified and confirmed by plating the inoculum as serial 10-fold dilutions on LB agar plates.

*Streptococcus uberis* strain PS10 and *Staphylococcus epidermidis* strain PS20 were isolated by the National Service for Udder Health and Milk Quality (NSUHMQ) laboratory from quarter milk samples originating from Israeli dairy cows affected by clinical mastitis. Bacteriological examination was performed according to National Mastitis Council guidelines (Shwimmer et al., 2007). Strains identification was further validated using 16S rRNA PCR analysis and sequencing as previously described (Wald et al., 2017). Strains were cryopreserved in 20% glycerol at −80°C until further processing. For IMM challenge, *Streptococcus uberis* and *Staphylococcus epidermidis* bacteria were grown on Heart Infusion Broth (238,400, HI, BD DIFCO) agar plates at 37°C for 24 h after which one isolated bacteria colony was transferred to 10 mL of HI broth for overnight growth at 37°C with shaking at 110 rpm. Pre-cultured bacteria were diluted 1:100 with fresh HI broth and grew to log phase for 3 h at 37°C with shaking at 110 rpm. Next, culture was centrifuged for 10 min at 4,000 × rpm and bacteria were washed twice with sterile PBS. Bacteria were resuspended in sterile PBS and inoculum was divided to 50 μL doses in 0.3 mL insulin syringes, approximately ~10^4^ CFUs and kept on ice until the IMM challenge. Challenge was quantified and confirmed by plating the inoculum as serial 10-fold dilutions on HI agar plates.

*Mycoplasma bovis* strain PG45 (ATCC 25523 / NCTC 10131) (Hale et al., 1962; Wise et al., 2011) was used in this study and prepared for challenge in lactating mice as previously reported by us (Schneider et al., 2022). Bacteria were propagated at 37^o^C in FF modified broth medium (Friis, 1975) supplemented with 0.5% (w/v) sodium pyruvate and 0.005% (w/v) phenol red. Stock cultures were grown to the titers of 10^8^–10^9^ CFUs/mL, aliquoted, and maintained at −80^o^C. For each stock, the number of CFUs per mL was determined by performing serial 10-fold dilutions in FF broth and by plating each dilution on agar in triplicates (Rodwell and Whitcomb, 1983); the agar plates were grown at 37°C, under an atmosphere of 5% CO2/95% air. For challenge, mycoplasma cultures were grown until mid-log phase and then harvested by centrifugation at 10,000 × *g* for 10 min at 4°C. The bacterial pellets were washed twice with the same volume of sterile PBS × 1 (Gibco1by Life Technologies) and centrifugated as described above. The bacterial cultures were resuspended in 500 μL of PBS to a final concentration of ~2 × 10^10^ CFUs/mL. The titers of *M. bovis* were calculated by plating serial 10-fold dilutions on FF agar.

### Murine mastitis model systems

Twelve to fourteen week-old female BALB/c mice were used in this project (Envigo, Israel). Intramammary challenge (IMM) was performed 8 days post-partum. Mice pups were removed before the challenge. Mice were first anesthetized (1.5–2.5% isoflurane in O_2_) and then challenged with 10^4^ CFUs of *E. coli* P4-NR, *Strep. uberis*, and *S. epidermidis* or with 10^9^ CFUs of *M. bovis* PG45. IMM infusion was performed through the teat canal in both L4 and R4 abdominal mammary glands (the fourth pair found from head to tail). Three mice/six glands were used in every experiment. Injections were conducted under a binocular using 0.3 mL insulin syringes with a 33-gauge blunt needle. Mice were sacrificed 24 h (*E. coli*, *Strep uberis*, and *Staph. epidermidis*) or 48 h (*M. bovis*) post challenge, and mammary tissues were harvested for histology, total RNA extraction and total bacterial counts. Glands harvested from normal non-challenged mice were used as controls.

### Bacterial counts and histological analysis

Mammary tissues were trisected for histology, total RNA extraction, and total bacterial counts as previously described (Mintz et al., 2019; Salamon et al., 2020). Harvested mammary tissues were weighed and homogenized in ice-cold PBS immediately after their removal and homogenates were plated as serial 10-fold dilutions on agar plates with and without antibiotics and bacterial colonies were counted following incubation at 37°C for 24 h to determine the output CFUs per 1 g of tissue. Specifically, *E. coli* was incubated on LB agar plates with and without chloramphenicol (25 μg/mL) for 24 h, *Streptococcus uberis* and *Staphylococcus epidermidis* were incubated on HI agar plates for 24 h, and *Mycoplasma bovis* was incubated on FF-modified broth medium agar plates for 3 to 5 days under an atmosphere of 5% CO_2_/95% air to determine the number of CFUs/g of tissue.

Samples for histological analysis were fixed in neutral buffered 4% PFA and embedded in paraffin (FFPE blocks), and sections were cut at a thickness of 5 μm and stained with hematoxylin and eosin (H&E) according to standard procedures. For immunofluorescent staining, FFPE sections were first incubated in a 70°C oven for 20 min, dewaxed in xylene and rehydrated by sequentially immersing slides in 100, 95, 80, and 70% ethanol, and then in DDW. Following rehydration, slides were placed in Tris/EDTA buffer, pH 9, for antigen retrieval (S236784-2, Agilent Dako) and boiled for 40 min. After cooling to room temperature, slides were washed twice in TBS-T solution (Tris-Buffered Saline, pH 7.6; Alfa Aesar, United Kingdom) with 0.1% Tween-20 (Fisher Scientific) and blocked by incubation in blocking solution containing 5% (v/v) donkey serum (Sigma) in TBS-T. FFPE sections were stained with DAPI (Sigma, Rehovot, Israel), and rat anti-mouse S100A9 (ab105472, Abcam), rabbit anti-mouse CD31 (Clone D8V9E, Ionpath), and rabbit anti-mouse Na/K-ATPase (Clone EP1845Y, Ionpath) primary antibodies. Next, sections were incubated with the following secondary antibodies; goat anti-rat IgG conjugated with Alexa Fluor 488 (a11006, Invitrogen), donkey anti-rabbit IgG conjugated with Alexa Fluor 647 (a31573, Invitrogen), and donkey anti-rabbit IgG conjugated with Alexa Fluor 694 (a21207, Invitrogen).

Fresh mammary tissues for fluorescence staining were fixed in 4% PFA overnight at room temperature, incubated with 15% (wt/vol) sucrose for 72 h at 4°C, and frozen in optimal cutting temperature compound (Sigma-Aldrich). Serial 15 μm cryosections were stained with DAPI and phalloidin (Sigma, Rehovot, Israel) and rabbit anti-human Ly6G (EPR3094; Abcam), rat anti-mouse ICAM-1 (Alexa Fluor 488 conjugated, 116,112, Biolegend), goat anti-mouse myeloperoxidase (AF3667, Biotest), rabbit anti-mouse Citrullinated histone H3 (ab5103, Abcam), rabbit ant-mouse ACKR1 (MBS7005916, MyBioSource), and rat anti-mouse CD90 (ab3105, Abcam) primary antibodies. Donkey anti-rabbit IgG conjugated with Alexa Fluor 546 (A10040, Sigma Thermo Fisher), and the above described secondary antibodies were used as described above. All immunofluorescence stainings were performed in blocking solution, and mounted with fluorescent mounting medium (S3023, Dako).

Pericytes were visualized in mammary tissues of transgenic mice expressing GFP under Nestin promoter (Mignone et al., 2004). Surplus tissues were kindly shared by Dr. Dalit Sela-Donenfeld, the Hebrew University. Telocytes were visualized in mammary tissues of transgenic mice expressing GFP under Foxol1 promoter (Shoshkes-Carmel et al., 2018). Surplus tissues were kindly shared by Dr. Michal Shoshkes-Carmel, the Hebrew University.

Microscopic analysis was performed using M1 Imager Axio epifluorescence microscope with an MRm Axio camera (Zeiss), and images were captured using Zen software V3.4.

### Real-time PCR and multiplex gene expression analysis

Lymph node divested L4 or R4 mammary glands were dissected from unchallenged and challenged mice and RNA was extracted for quantitative real-time PCR (QPCR) and Nanostring nCounter profiling. The qPCR analysis of mammary tissues was performed as previously described by us (Salamon et al., 2020). Briefly, total RNA was isolated from mammary tissue using the GeneElute Mammalian Total RNA Miniprep Kit (Sigma-Aldrich, Rehovot, Israel) combined with on-Column DNase I Digestion Set (Sigma). Reverse transcription was performed using qScript cDNA Synthesis Kit (Quanta BioSciences, Gaithersburg, MD, United States) and cDNA was used for subsequent qPCR reactions. PCR was conducted on a StepOne Plus PCR instrument (Applied Biosystems) using the FAST qPCR Universal Master Mix (Kappa Biosystems, Boston, MA, United States). All reactions were performed in triplicates and the gene expression levels for each amplicon were calculated using the ΔΔCT method (Livak and Schmittgen, 2001) and normalized against Ptma mRNA. Melting curve analysis was performed on each primer set to confirm amplification of a single product and all amplicons were sequenced to ensure reaction specificity (data not shown).

Using 100 ng of RNA per sample, RNA was profiled using Nanostring nCounter Mouse Myeloid Innate Immunity V2 Panel of 754 genes, including 20 internal reference genes for data normalization.^1^ Data was analyzed using nSolver™ analysis software version 4.0, GraphPad, and Excel softwares. Differential expression analysis was performed with *p*-values adjusted for multiple comparisons according to Benjamini and Hochberg, corresponding to a false discovery rate (Benjamini and Hochberg, 1995). Differentially expressed gene sets were mapped onto gene interaction networks from STRING v.11 (Szklarczyk et al., 2018).

### Gene Set Enrichment Analysis (GSEA)

Gene Set Enrichment Analysis (GSEA) (Subramanian et al., 2005) was done using the java-based GSEA program and curated molecular signature database (MSigDB) both provided by the BROAD institute.^2^ The Hallmark gene set of 50 well-defined biological states or processes (Liberzon et al., 2015), c5GOBP gene set (MSigDB; c5 Gene ontology Biological Processes), and neutrophil extracellular trap formation gene set taken from Wu et al. (2020) were used to assess the effect of bacterial challenge on murine mammary transcript expression.

### Ligand-receptor analysis

Bulk gene expression data of 734 murine immune genes was produced using Nanostring nCounter technology. Ligand-receptor communication scores were calculated using the expression correlation method as previously described by Armingol et al. (2021). For comprehensive systematic analysis of putative ligand-receptor interactions, we used CellTalkDB (Shao et al., 2020) which is a manually curated database of literature-supported ligand-receptor interactions in human and mouse. The communication score (CS) was computed for every ligand-receptor pair in the gene expression data. Correlation coefficients were calculated as CS for each ligand-receptor pair across all data samples. We assumed that a communication pathway is active only when ligand and receptor were both significantly (adjusted *p*-value <0.05) differentially expressed with fold change (FC) greater than 1 (see representation of data analysis using Volcano and correlation plots in Supplementary Figure 1). Ligand-receptor interactions and CS were visualized using heatmaps (GraphPad) and Sankey diagrams (SankeyMATIC).^3^

## Results

### Establishment of murine mastitis model system

**Figure 1:**
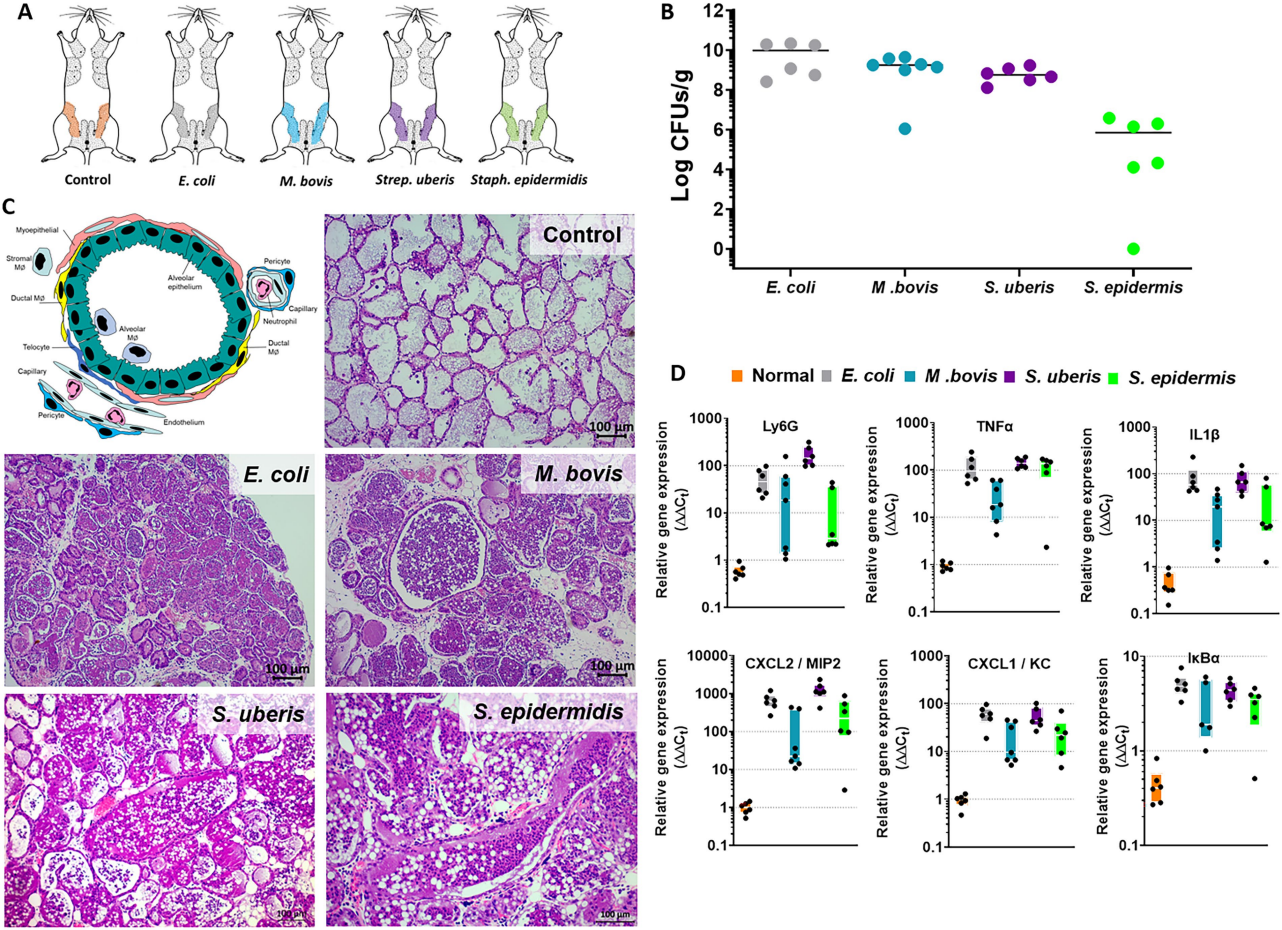
The murine mastitis model system. Lactating 6–8 weeks old BALB/c mice were infected with live *E. coli* P4-NR, *M. bovis* PG45, *Strep. uberis* PS10 and *Staph. epidermidis* PS20 bacteria by intramammary infusion through the teat canal **(A)**. Mice were humanely sacrificed after 24 or 48 h (*M. bovis* only) and mammary tissues were processed for total bacterial counts **(B)**, histology (H&E staining of FFPE tissue sections in **C**) and QPCR analysis **(D)**. Annotated schematic diagram of mammary alveolar structural and immune cell elements is shown in **C** (left top panel). Diseased glands were characterized by bacterial counts **(B)**, massive recruitment of blood neutrophils into the alveolar and ductal spaces **(C)** also indicated by increased relative expression of the neutrophil marker Ly6G **(D)**. Mammary inflammation was associated with increased expression of TNFɑ, IL1β, CXCl1, CXCL2, and IkBɑ **(D)**. Scale bars; 100 μm.

Experimental mastitis model systems were established in lactating BALB/c mice using intramammary (IMM) infusion of bacterial suspension to glands L4 and R4 (Figure 1A). We have used *E. coli* strain P4-NR and the type strain *M. bovis* PG45 as previously reported by us (Salamon et al., 2020; Schneider et al., 2022). Field strains of *Strep. uberis* (strain PS10) and *Staph. epidermidis* (strain PS20), isolated from clinical mastitis in dairy cows, are reported here for the first time. The disease in lactating mice was characterized by high bacterial counts in the gland tissue homogenates (Figure 1B), massive recruitment of blood neutrophils into tubular and alveolar milk spaces (Figure 1C), which is further supported by increased expression of the neutrophil marker Ly6G (Figure 1D), and increased expression of inflammatory markers such as Tnfɑ, Il1β, Cxcl1 (KC), Cxcl2 (Mip2), and IkBɑ (Figure 1D).

**Figure 2:**
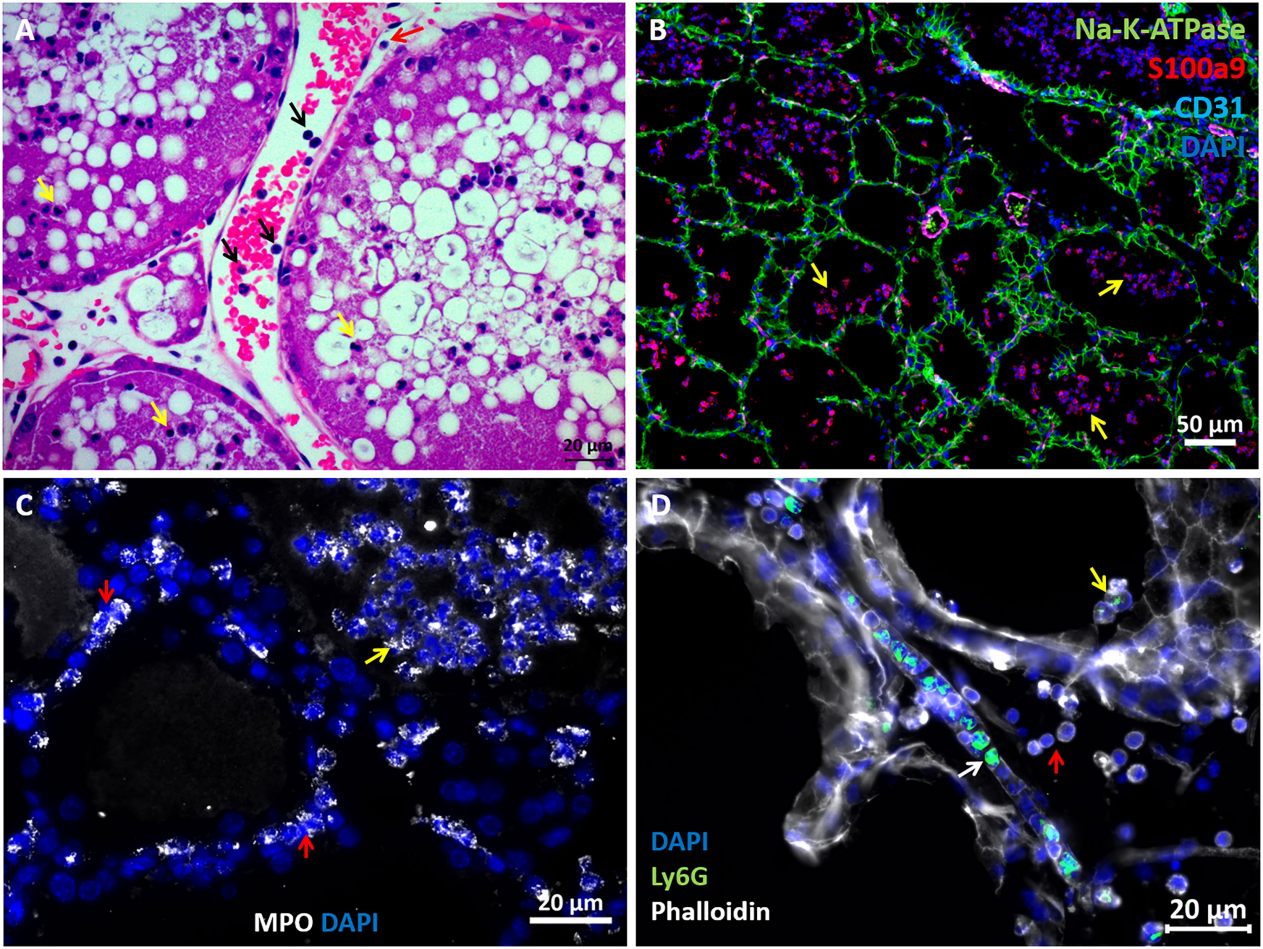
Visualization of neutrophil recruitment in infected mammary glands. H&E staining of FFPE section showing post capillary venule adjacent to the alveoli **(A)**. Blood neutrophils are visible with RBCs and adherent to the endothelium (black arrows in **A**). Neutrophils are also visible in the parenchyma (red arrow in **A**) and alveolar space (yellow arrows in **A**). Fluorescence staining of cryosections from infected glands using DAPI (blue in **B–D**), anti Na-K-ATPase (green in **B**), S100a9 (red in **B**), CD31 (purple in **B**), myeloperoxidase (MPO, white in **C**) and Ly6G (green in **D**) antibodies, and phalloidin (white in **D**). Mammary epithelial cells are demarcated with anti Na-K-ATPase (green in **B**) or phalloidin (white in **D**). Neutrophil (S100a9^+^, MPO^+^, and Ly6G^+^) are visible in blood vessels (white arrow in **D**), parenchyma (red arrows in **C**) and alveoli (yellow arrows in **B–D**). Individual channels merged in **B** are shown in Supplementary Figure 2. Scale bars; 20 μm **(A, C,D)** and 50 μm **(B)**. Representative images from mammary glands 24 h after IMM challenge with *E. coli* P4-NR bacteria.

**Figure 3:**
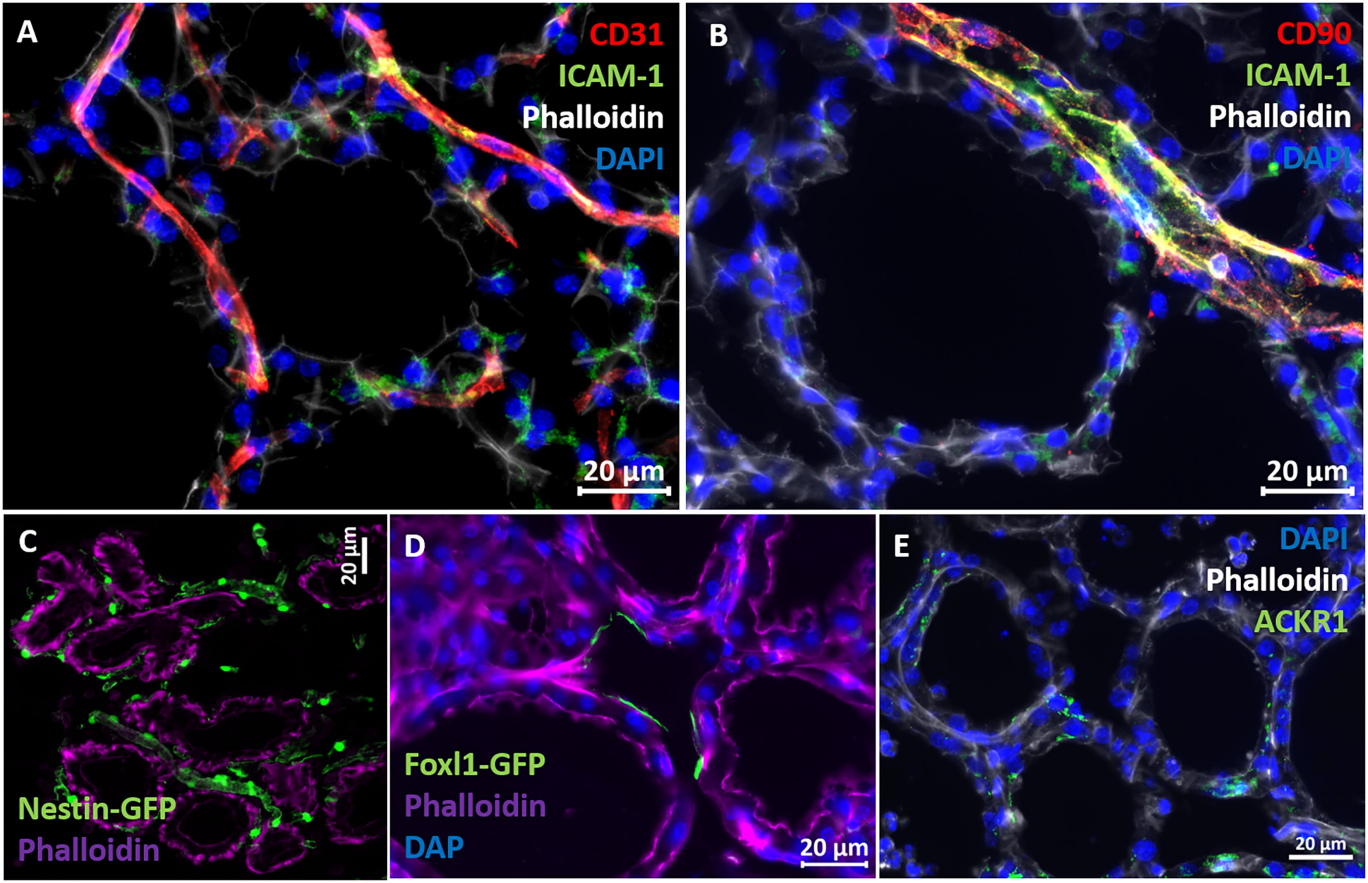
Characterization of vascular structures enveloping the alveoli in normal murine mammary gland. Capillaries **(A)** and post capillaries venules **(B)** were demonstrated using the pan-endothelial marker CD31 **(A)** and pericyte marker CD90 **(B)**. Cell adhesion protein ICAM-1 is expressed by endothelial cells and pericytes **(A,B)**. Expression of the post capillary venules marker ACKR1 is visualized in **C**. Representative sections of cryosections stained with DAPI, phalloidin, and fluorescent anti-CD31 **(A)**, anti-ICAM1 **(A,B)**, anti-CD90 **(B)**, and anti-ACKR1 antibodies **(E)**. Pericytes were further demonstrated using normal mammary tissues obtained from Nestin-GFP transgenic mice **(C)**, and telocytes were demonstrated using mammary tissues obtained from normal Foxpl1-GFP transgenic mice **(D)**. Scale bars; 20 μm.

The above described murine mastitis model systems represent the most prevalent mammary pathogenic bacteria in dairy animals; Gram-negative coliforms, Gram-positive streptococci and staphylococci, and mycoplasma. The shared hallmark of the disease caused by acute mammary infection by all these pathogens is massive recruitment of blood neutrophils into the tubular and alveolar milk spaces (Figures 1C,D). Neutrophil trafficking requires transendothelial, parenchymal, and transepithelial neutrophil migration. The structural cellular elements putatively involved in this process are schematically outlined in Figure 1C. Using immunofluorescence staining for S100a9, myeloperoxidase (MPO), and Ly6G as neutrophil markers, we demonstrate here neutrophils at each of the above stages of recruitment (Figure 2 and Supplementary Figures 2, 3). The immensely arborized alveolar and tubular epithelial duct system of the lactating mammary gland is surrounded by elaborative honeycomb-like vascular system in close proximity to all epithelial elements (Supplementary Figure 2C). The site of neutrophil extravasation and the underlying molecular mechanisms involved are still unknown in the mammary gland. In mice, the capillaries branch from approximately 10 μm lumen diameter vessels to reach an average of <6 μm diameter, then converge into larger post-capillary venules with diameter > 6 μm, that project to ascending venules (Fujiwara and Uehara, 1984; Kucharz et al., 2021). Based on size and the pan-endothelial cell marker, CD31, the mammary vascular system can be visualized and identified (Figures 2B, 3A and Supplementary Figure 2C). Moreover, as previously reported, we show here steady state expression of the adhesion molecule ICAM-1 by these blood vessels (Figures 3A,B), and their surrounding networks of pericytes (Nestin^+^ and CD90^+^) and telocytes (Foxl1^+^) (Figures 3C,D; Fujiwara and Uehara, 1984; Gherghiceanu and Popescu, 2005; Klein et al., 2022). Post capillary venules, which are considered a specialized site of immune cells recruitment, were demonstrated using the ACKR1 marker (Figure 3E; Thiriot et al., 2017; Girbl et al., 2018).

### Immune genes response elicited by mastitis pathogens

**Figure 4:**
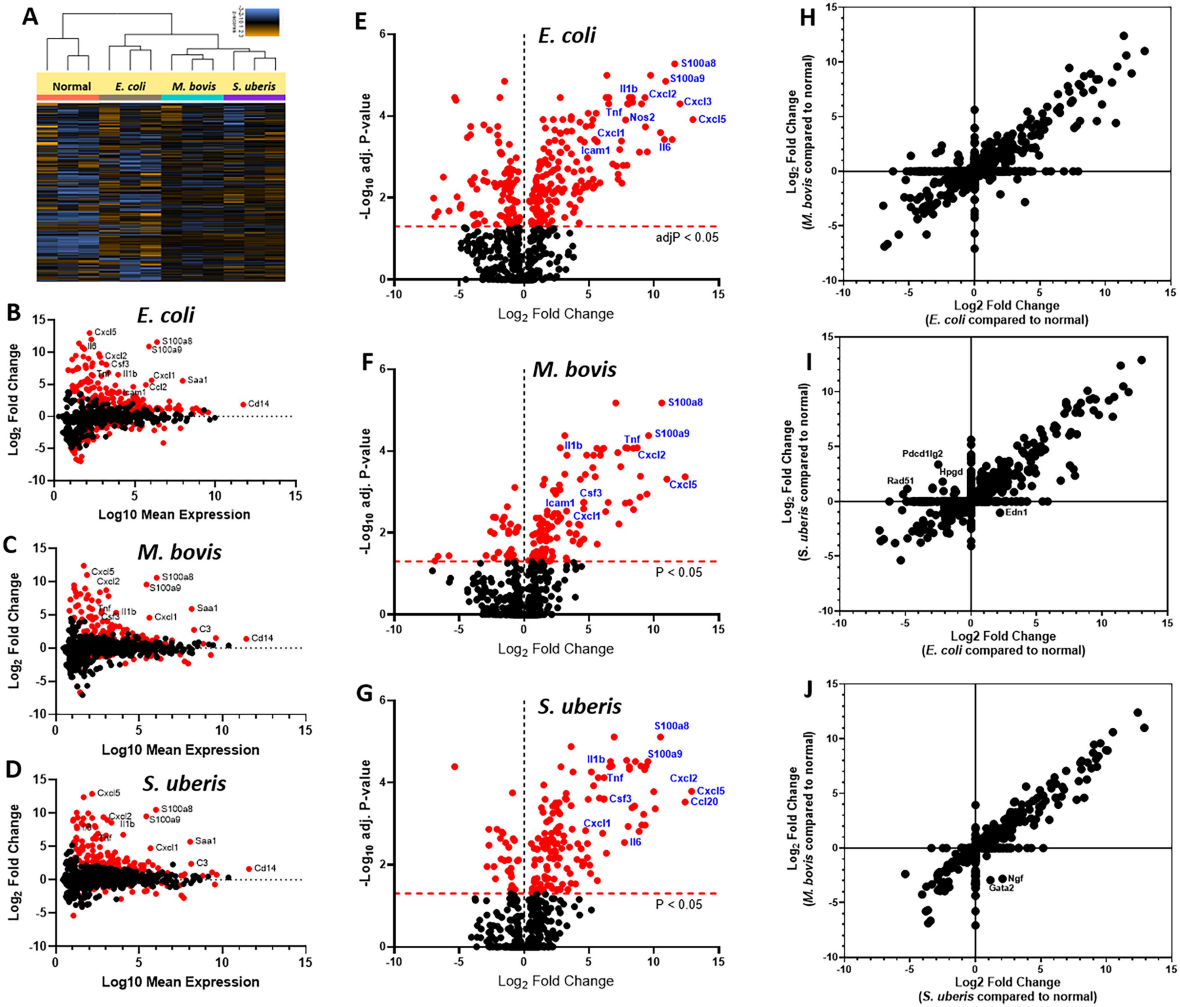
Mammary immune profiling in experimental murine mastitis using multiplex gene expression analysis. Lactating BALB/c mice were challenged by intramammary infusion of *E. coli* P4-NR, *M. bovis* PG45, and S*trep. uberis* PS10 bacteria and total RNA was extracted from mammary tissues and profiled using Nanostring nCounter Mouse Myeloid Innate Immunity V2 Panel which includes 754 genes. Following quality check and normalization, expression data presented using cluster analysis **(A)**, MA plots **(B–D)**, Volcano plots **(E–G)**, and correlation plots **(H–J)**. MA plots showing comparison of gene expression between mammary glands challenged with *E. coli* **(B)**, *M. bovis* **(C)**, or *Strep. uberis* **(D)** and normal control glands. Data represent the combined analysis of three biologically independent samples. The *x*-axis represents the log10 of mean expression in normal and challenged glands. The *y*-axis indicates the differences in gene-expression level between infected and non-infected glands (log2 fold change), larger positive values represent genes with higher expression in infected glands relative to the normal glands and larger negative values represent genes with higher expression in the normal glands relative to the infected glands. Significantly expressed genes (adjusted *p*-value < 0.05) are highlighted in red, and selected genes are annotated. Volcano plots **(E–G)** showing fold-change of gene expression in infected glands compared to non-infected normal control glands. The *x*-axis indicates the log2 differences in gene-expression level between infected and non-infected glands (*y*-axis in MA plots). The *y*-axis shows the –log10 of the adjusted *p* values for each gene, with larger values indicating greater statistical significance. Those genes showing significantly different expression between infected and normal groups are highlighted in red and the dashed horizontal red line marks the cut-off for p-value lower than 0.05 corresponding to these *p*-values on the *y*-axis. Black points represent genes for which there was no significant difference in gene expression. Correlation plots **(H–J)** present all significantly changed genes (*P*adj-value <0.05; |log2 fold-change| >1) in either challenged gland, while non-significant log2 fold-changes are assigned the value of 1 and are located on the *x*-axis or on the *y*-axis (log2 = 0).

**Figure 5:**
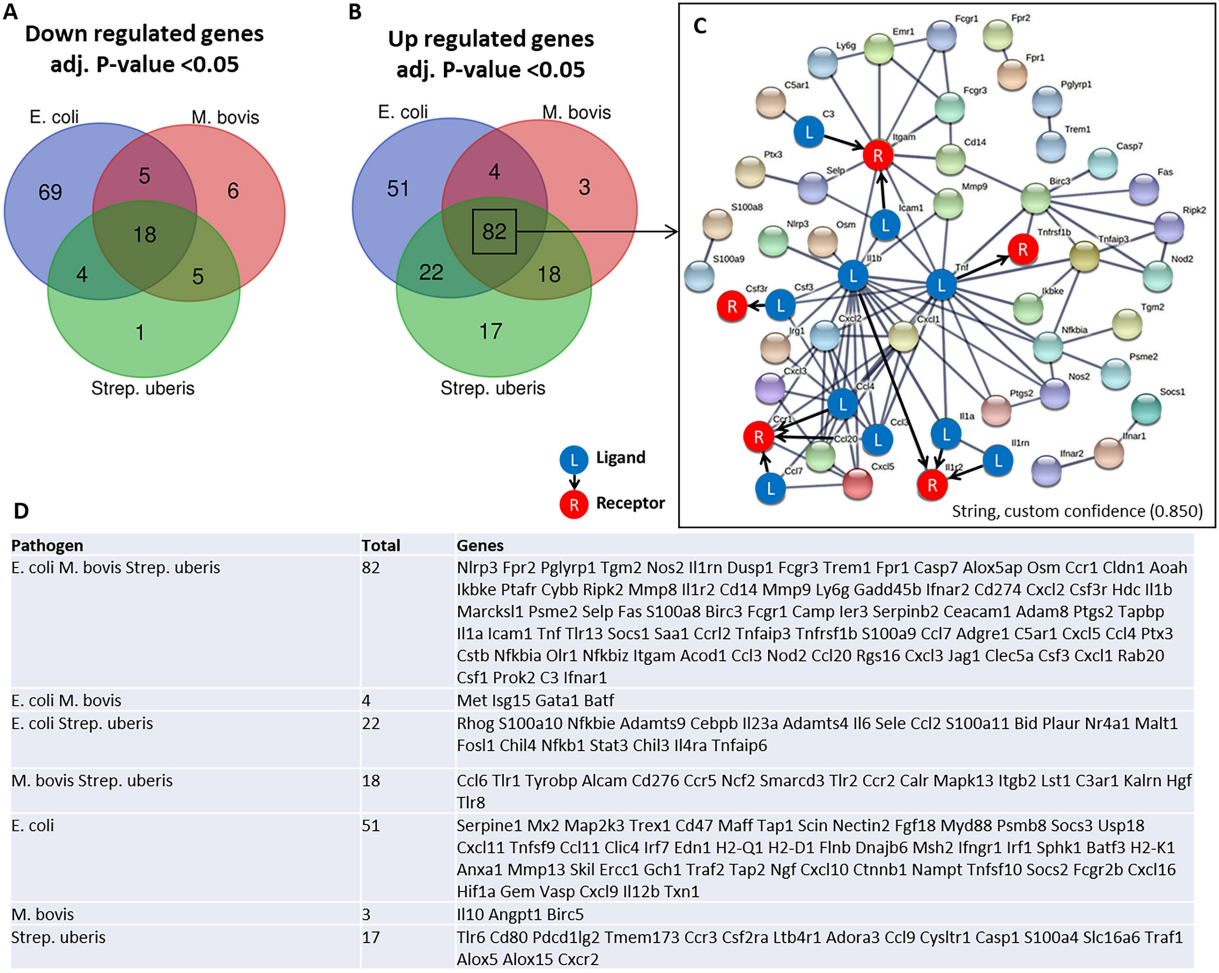
Venn diagram analysis of down regulated **(A)** and up regulated **(B,D)** differentially expressed genes was used to analyze the similarities and differences in mammary gene response following challenge with *E. coli*, *M. bovis*, and *Strep. uberis* bacteria. Genes significantly (adjusted *p*-value <0.05) upregulated following challenge by all three pathogens were further analyzed using STRING demonstrating physical and functional interactions of putatively expressed proteins (82 common genes in **C**). The receptor (R) and their ligands (L) interactions were based on CellTalkDB database. STRING (https://string-db.org/cgi/), custom confidence; 0.850, line thickness indicates the strength of data support.

To better understand the molecular mechanisms underlying the above described inflammatory process and neutrophil recruitment, we next performed immune profiling of mammary glands challenged with *E. coli*, *M. bovis*, and *Strep. uberis* bacteria. These strains were chosen as representatives of mammary pathogenic Gram-positive, Gram-negative, and mycoplasma species, while *Staph. epidermidis* was not further analyzed in this study. We used the Nanostring nCounter technology to profile the expression of 734 immune genes in challenged glands in comparison with normal unchallenged glands. Total and differential gene expression (log_2_ fold-changes) in *E. coli*, *M. bovis*, or *Strep. uberis* challenged glands are presented and compared in Figure 4. Cluster analysis showed higher similarity in gene expression following challenge with *M. bovis* and *Strep. uberis* than following *E. coli* challenge (Figure 4A). MA (Figures 4B–D) and Volcano (Figures 4E–G) plots present normalized expression and relative expression for each challenge organism. In the *E. coli* challenged glands, 255 genes (159 upregulated and 96 downregulated) were significantly differentially expressed in comparison with the normal unchallenged glands (adjusted *p*-value <0.05). In the *M. bovis* challenged glands, 141 genes (107 upregulated and 34 downregulated) were significantly differentially expressed in comparison with the normal unchallenged glands (adjusted p-value <0.05). In the *Strep. uberis* challenged glands, 167 genes (139 upregulated and 28 downregulated) were significantly differentially expressed in comparison with the normal unchallenged glands (adjusted p-value <0.05). Next, we used correlation scatter plots (Figures 4H–J) and Venn diagram analysis (Figures 5A,B) of upregulated and down regulated differentially expressed genes to analyze the similarities and differences in mammary immune gene response to each pathogen. Scatter plots present all significantly changed genes (*P*_adj_-value <0.05; |log_2_ fold-change| >1) in either challenged gland, while non-significant log2 fold-changes are assigned the value of 1 and are located on the *x*-axis or on the *y*-axis (log2 = 0 in Figures 4H–J). Taken together, our analysis revealed a core of 100 genes which are similarly regulated in *E. coli*, *M. bovis*, and *Strep. uberis* challenged glands (Figures 5A–D) and based on STRING analysis functionally and/or physically interacting (Figure 5C) in response to the challenge with each pathogen.

### Gene Set Enrichment Analysis (GSEA)

**Figure 6:**
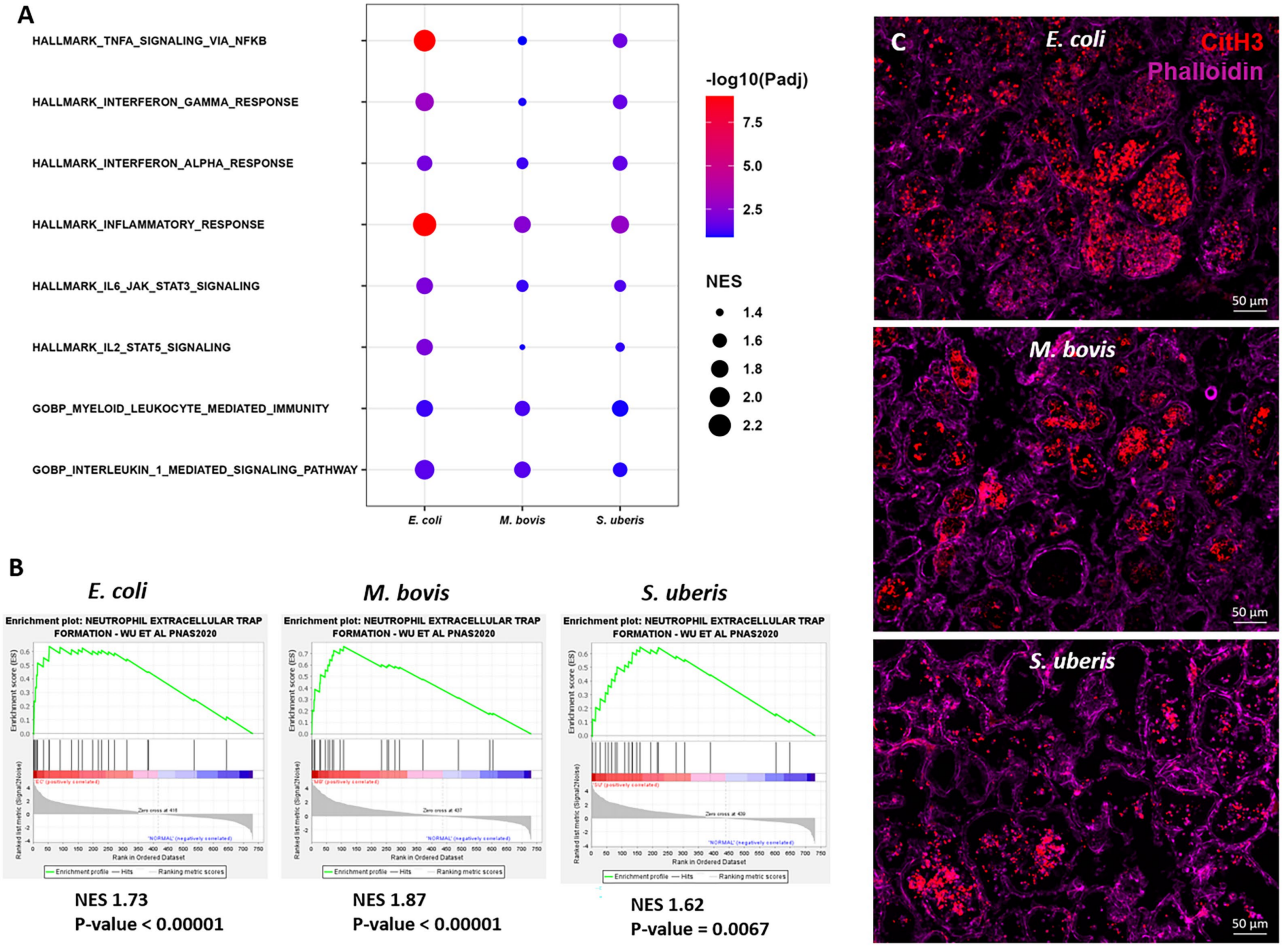
Gene set enrichment analysis (GSEA) of mammary gene expression data using HALLMARK and c5GOBP gene sets **(A)**, and neutrophil extracellular trap (NET) gene set **(B)** comparing infected glands to normal controls. The most enriched HALLMARK and c5GOBP gene sets are presented for *E. coli*, *M. bovis*, and *Strep. uberis* **(A)**. Gene set enrichment plots (Mountain plots in **B**) showing upregulation of genes associated with NET formation in infected glands. NES, normalized enrichment score. NET GSEA was further validated using immunofluorescence staining for citrullinated histone 3 (CitH3 in **C**). Representative images of mammary tissue following challenge with *E. coli* (top panel in **C**), *M. bovis* (middle panel in **C**) and *Strep. uberis* (bottom panel in **C**). Epifluorescence microscopic images of phalloidin and anti-CitH3 antibody staining suggesting alveolar neutrophils forming NETs. Scale bars; 50 μm.

To further explore the immune processes associated with these differentially expressed genes, we next performed Gene Set Enrichment Analysis (GSEA) of our expression data comparing bacterial infections to control samples (Figure 6). Significantly enriched gene sets common to the three pathogens are presented in Figure 6A and include TNFɑ signaling *via* NFkB, Interferon gamma and alpha response, IL1-mediated signaling, myeloid leukocyte response, and IL6-JAK-STAT3 signaling. Moreover, since neutrophil extracellular trap (NET) genes set is not available in MSigDB, we created a new gene set based on previously published data (Wu et al., 2020). GSEA revealed significant enriched expression of genes associated with NET in glands challenged by all three pathogens (Figure 6B). This finding was further validated using immunofluorescence staining for citrullinated histone 3 (CitH3; Figure 6C), MPO and DNA staining (Supplementary Figure 4) of mammary tissues infected by each of the three pathogens. CitH3, combined with MPO and DNA staining, is a specific marker of neutrophils forming NET (Radermecker et al., 2022; Sanders et al., 2022). We show here that some, albeit not all, neutrophils recruited from blood into the alveolar and tubular milk spaces form NET in response to IMM infection with *E. coli*, *M. bovis*, and *Strep. uberis*.

### Receptor-ligand analysis

**Figure 7:**
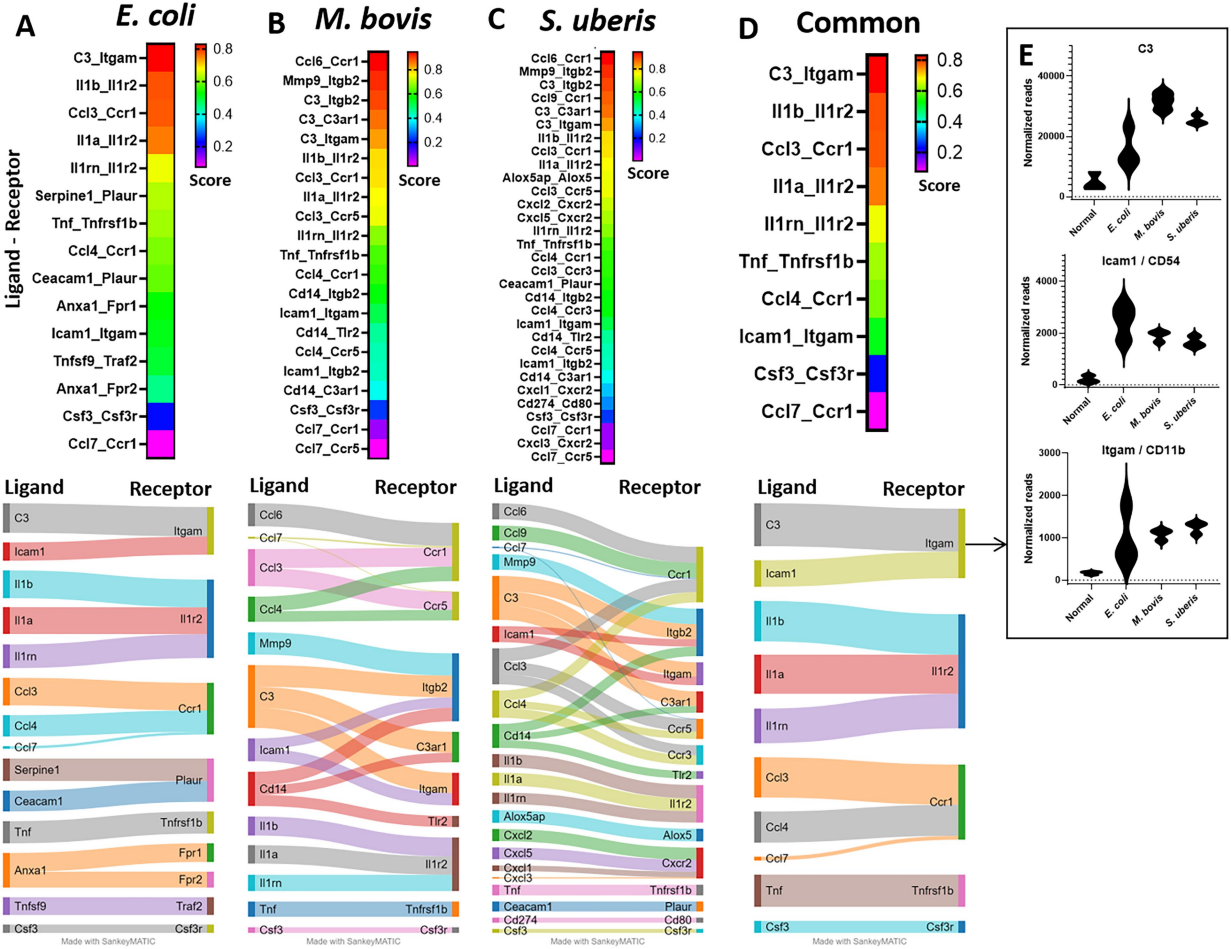
Ligand-receptor analysis in *E. coli* **(A)**, *M. bovis* **(B)**, and *Strep. uberis* **(C)** murine mastitis and common for all three pathogens **(D)**. Mammary tissues were harvested following intramammary (IMM) bacterial challenge, bulk RNA was extracted and gene expression of 734 immune genes was quantified using Nanostring nCounter technology. Ligand-receptor data were obtained from the CellTalkDB database and communication score (CS) was computed for every ligand-receptor pair. CS Correlation coefficients were calculated for each ligand-receptor pair across all data samples and were used as CS for every ligand-receptor pair in the gene expression data. We assumed that a communication pathway is active only when ligand and receptor were both significantly (*P*adj <0.05) differentially expressed with fold change (FC) greater than 1 (see Supplementary Figure 1). Top (CS > 0.2) ligand-receptor interactions were visualized using heatmaps and Sankey diagram. Normalized expressions of the top common R-L pairs; C3 and Icam-1 with Itgam, are presented in **E**.

To better understand the molecular mechanisms unveiled by our expression data, we applied computational analysis of ligand-receptor (L-R) gene pairs (Figure 7). While many putative L-R interactions were pathogen-specific (Figures 7A–C), our analysis revealed interactions shared by the three pathogens (Figure 7D). These include interaction of the cytokines IL1β, IL1ɑ, TNFɑ, and Csf3 with their receptors (Il1r2, Tnfrsf1b, and Csf3r, respectively), chemokines CCL3 and CCL4 (with CCR1), and complement C3 and ICAM1 (with Itgam). Normalized gene expressions of C3, Icam1, and Itgam (Cd11b) are presented in Figure 7E. The high expression of ICAM-1 in blood vessels of infected mammary glands was further validated using immunostaining and epifluorescence microscopy (Supplementary Figure 5).

## Discussion

Mastitis in dairy animals is a prevalent and economically important disease. The disease is caused by ascending bacterial infection through the teat canal and *E. coli*, *M. bovis*, *Strep. uberis*, and *Staph. epidermidis* (coagulative-negative staphylococcus) are common etiologies. Studying the disease mechanism is difficult in dairy animals and to this end, murine mastitis models are useful experimental platforms. Although murine mastitis studies using the above-mentioned pathogens were performed for many years (Anderson et al., 1976; Anderson, 1983; Sordillo and Nickerson, 1986; Breyne et al., 2015), current development of genetic and analytic technologies supersedes all those used in previous studies. The advantages and limitations of the murine mastitis model system were eloquently reviewed and discussed by Ingman et al. (2015). We present here a panel of murine mastitis models in lactating mice infected with mammary pathogenic bacteria cultured from field cases of clinical mastitis in dairy cows. Histopathological analysis of the glands shows an undistinguishable inflammatory response characterized by massive recruitment of blood neutrophils into alveolar and tubular milk spaces. As previously showed (Breyne et al., 2015), no changes were observed in control glands infused with the aqueous carrier of the bacteria and therefore not included in the current analysis. These acute pathological changes recapitulate the disease processes observed in field cases and underscore the translational utility of the murine model systems. Moreover, the transcriptional response observed in our model systems is highly similar to those reported in numerous studies in dairy animals (Younis et al., 2016; Rainard et al., 2022). It is generally accepted that mastitis is a MAMP-mediated disease and that specialized bacterial virulence factors other than fitness factors that enable initial growth and colonization are unlikely to play a role in acute mastitis (Keane, 2019; Salamon et al., 2020). Our aim was to develop experimental platforms and research tools that will enable us to decipher the molecular mechanisms activated by MAMPs of diverse mammary pathogens yet culminating uniformly in the recruitment of blood neutrophils into milk spaces. To better understand the mechanisms of neutrophil recruitment, we show here the structural architecture of neutrophil trafficking from capillaries and post capillary venules which are surrounded by a network of pericytes and telocytes. The role pericytes and telocytes play in neutrophil recruitment to the mammary gland is currently unknown; however, transgenic mice presented in our study can expedite this avenue of research.

MAMPs released by colonizing bacteria activate MAE cells and alveolar macrophages, causing them to release inflammatory mediators, chemo attractants, and chemokines which are the enablers of neutrophil trafficking (Rainard et al., 2022). Our gene expression results are well corroborated with previous *in vitro* and *in vivo* studies showing that activation of mammary inflammation by MAMPs is mediated by TNFɑ, IL-1β, and the NFkB and IL6-JAK-STAT3 pathways (Kobayashi et al., 2013; Sharifi et al., 2018; Weller et al., 2019; Passe Pereira et al., 2021). Activation of the above pathways leads to increased production of chemokines which function as chemo attractants triggering neutrophil trafficking. In our challenge studies, the three pathogens significantly increased the expression of the chemokines Cxcl1 (KC), Cxcl2 (Mip2), Cxcl3, Cxcl5, Ccl3, Ccl4, and Ccl7. Neutrophil recruitment occurs through an adhesion cascade, which consists of several steps. Initially, neutrophils are slowed by selectin-mediated interactions, which allows for chemokine-induced activation of integrins, firm adhesion, and transmigration across the endothelial layer. Indeed, we show here that the expression the Selp gene encoding P-selectin was significantly increased in mammary tissues infected by each of the three pathogens. The Itgam gene encodes the protein CD11b which together with CD18 (encoded by Itgb2) forms the ɑMβ2 integrin MAC-1. The Itgam-Icam1 pair was scored high in our L-R analysis and we also showed the expression of ICAM-1 on the mammary vasculature. These data suggest that MAC-1 which is constitutively expressed on blood neutrophils enables adherence of neutrophils to endothelial surface and transendothelial migration. Following transendothelial migration, neutrophils en route to the alveolar space need to transverse the parenchyma and epithelial cell layer. We can only speculate that the upstream of inflammatory mediators originating from the mammary epithelial cells and macrophages keep them on track. Moreover, transversing the alveolar epithelial cell layer might also involve CD11b/CD18 interactions as previously reported in other epithelial lined organs (Azcutia et al., 2023).

The highest common L-R score obtained in our analysis was for C3-Itgam interaction. As we show here and previously reported by others, C3 is highly expressed in the murine and bovine lactating mammary gland and expression is considerably increased in response to mammary infection (Salamon et al., 2020). Moreover, we have previously showed that while elimination of downstream activation and degradation products of C3 (using cobra venom factor treatment) regained mammary fitness of rough strains of *E. coli*, inflammation was not affected (Salamon et al., 2020). Taken together, we can further speculate that there is considerable redundancy in the molecular mechanisms underlying remote activation of neutrophil trafficking in the infected mammary gland and that C3 protein has important roles in this process irrespective of its activation and degradation products. Previous studies, including single-cell RNA sequencing of the mouse mammary gland, showed that C3 can be expressed and produced by many cell types, including epithelial and myoepithelial cells, macrophages and fibroblasts (Ricklin et al., 2016; Bach et al., 2017; Li et al., 2020).

For now, we can only speculate that many of the upregulated genes observed in our analysis translate into components of the above described process. As previously reported in other tissues (Hyun and Hong, 2017; Girbl et al., 2018; Sekheri et al., 2021), CXCL1 (KC), CXCL2 (MIP2), ICAM-1, ACKR1, Selp (P-selectin), and Itgam (Cd11b), are all most probably involved in transendothelial neutrophil trafficking in mastitis. Moreover, the chemokine receptor CCR1 and its ligands CCL3 and CCL4, unveiled by our L-R analysis, were previously reported as amplifiers of neutrophil recruitment (Hyun and Hong, 2017). However, gene expression data and pathway analysis need to be validated using protein imaging and intervention studies. For example, our NET gene set analysis was validated by immunofluorescence imaging for the NET marker citrullinated H3 in alveolar neutrophils. However, the relevance of NET formation in the pathogenesis of the disease needs to be evaluated using specific chemical inhibitors and/or genetic manipulation of certain genes in neutrophils. Further validation in dairy animals will be even more difficult. Other interesting pathways that were significantly upregulated by the three pathogens were interferon alpha and gamma response. Noteworthy is a recent single-cell RNA sequencing study showing similar response in recruited inflammatory neutrophils (Grieshaber-Bouyer et al., 2021); moreover, this response might even be related to NET formation (Grunwell et al., 2020; Moreira-Teixeira et al., 2020; Peng et al., 2022). However, the mechanism behind interferon-related transcripts expression and their role in neutrophil function and immune response remains to be determined.

In conclusion, we established robust and reproducible murine mastitis model systems using important and diverse mammary pathogenic bacteria. Using a newly developed technology, we analyzed the immune genes response to mammary infection in these model systems. We show here that mammary infection with *E. coli*, *M. bovis*, and *Strep uberis* resulted in activation of conserved core of immune genes and pathways including NET formation. Important limitations of this study were lack of temporal and spatial analysis of disease dynamics. Combining these model systems with current spatially-resolved multi-omics technologies will greatly enhance our understanding of the biology of inflammation in the mammary gland.

## Data availability statement

The data presented in the study are deposited in the Gene Expression Omnibus (GEO) repository (link: https://www.ncbi.nlm.nih.gov/geo/), accession number GSE225127.

## Ethics statement

The IACUC approvals were obtained prospectively from the Ethics Committee for Animal Experimentation, the Hebrew University of Jerusalem.

## Author contributions

NS and IL conceived and designed the research. PS and EN-E performed the experimental work. NW performed immunostainings. HS assisted in data analysis and writing of the manuscript. NS took the lead in writing the manuscript. All authors provided critical feedback and helped shape the research, analysis, manuscript, read, and approved the final manuscript.

## Funding

This research was funded by US-Israel Binational Agricultural Research and Development fund (BARD), grant number IS-5066-18R and by the Chief Scientist, Ministry of Agriculture and Rural Development, grant number 33-08-0006.

## Conflict of interest

The authors declare that the research was conducted in the absence of any commercial or financial relationships that could be construed as a potential conflict of interest.

## Publisher’s note

All claims expressed in this article are solely those of the authors and do not necessarily represent those of their affiliated organizations, or those of the publisher, the editors and the reviewers. Any product that may be evaluated in this article, or claim that may be made by its manufacturer, is not guaranteed or endorsed by the publisher.

## Footnotes

^1^ https://www.nanostring.com/products/PanelPro

^2^ https://www.broadinstitute.org/gsea/index.jsp

^3^ https://sankeymatic.com/
