# Supplementary material for: Immune profiling of experimental murine mastitis reveals conserved response to mammary pathogenic Escherichia coli, Mycoplasma bovis, and Streptococcus uberis
Source: Front Microbiol. 2023 Mar 24;14:1126896. doi: 10.3389/fmicb.2023.1126896 (PMC10080000; doi:10.3389/fmicb.2023.1126896)
Supplement: Supplementary file 1 [file Data_Sheet_1.docx]

Supplementary Material

**Immune profiling of experimental murine mastitis reveals conserved response to mammary pathogenic *Escherichia coli*, *Mycoplasma bovis* and *Streptococcus uberis***

Peleg Schneider, Hagit Salamon, Nathalie Weizmann, Einat Nissim-Eliraz, Inna Lysnyansky and Nahum Y. Shpigel*****

*** Correspondence:** Corresponding Author: [nahum.shpigel@mail.huji.ac.il](mailto:nahum.shpigel@mail.huji.ac.il)

## Supplementary Figures


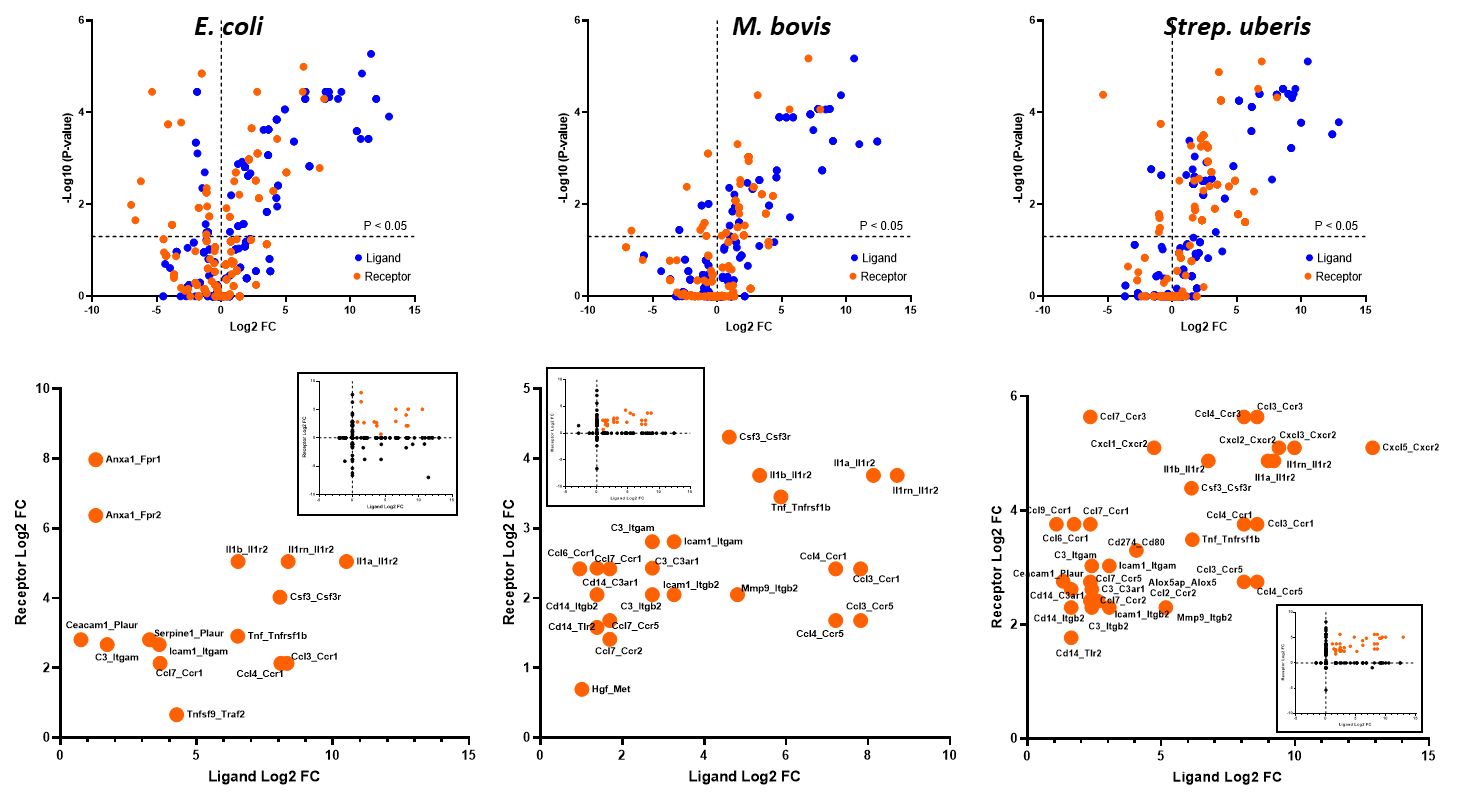


**Supplementary Figure 1.** Data analysis and computational steps used for the calculation of ligand-receptor communication scores for genes in the expression data set. Ligand-receptor (L-R) data was obtained from the CellTalkDB data base (http://tcm.zju.edu.cn/celltalkdb/index.php). All ligands (blue dots) and receptors (red dots) identified in the data set are presented using a Volcano plot for each pathogen (top panels). We assumed that a communication pathway is active only when ligand and receptor were both significantly (adjusted P-value < 0.05) differentially expressed with fold change (FC) greater than 1 (all data points above the horizontal broken lines in the Volcano plots). Significantly differentially expressed L-R pairs are presented for each pathogen in the annotated correlation plots in the bottom panels. Insert correlation plots present all L-R pairs, black dots represent L-R pairs that were excluded from the analysis.


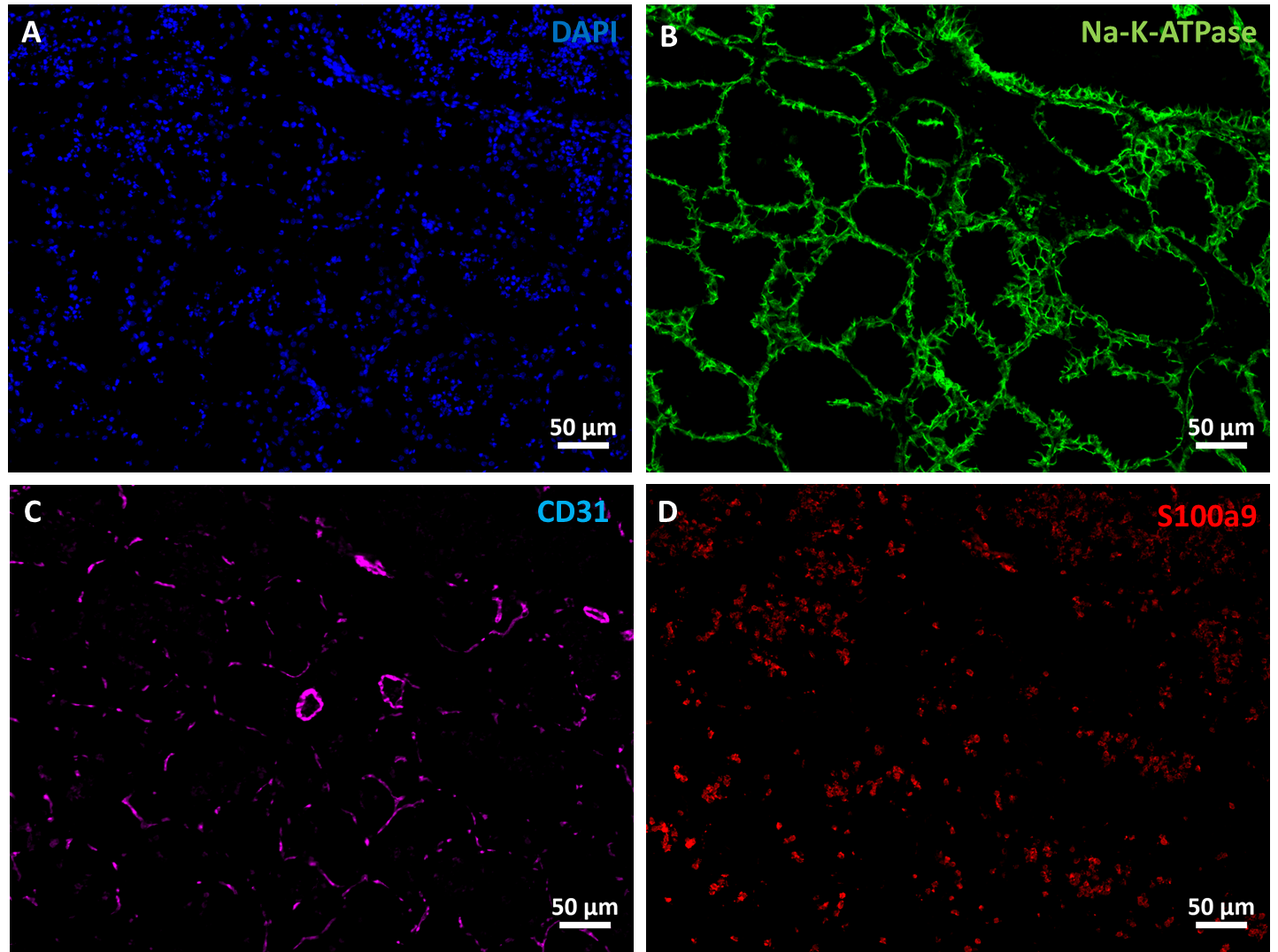


**Supplementary Figure 2**. Individual channels of merged image Figure 3B. Epifluorescence microscopic images of infected mammary gland stained using DAPI (A), anti Na-K-ATPase (B), anti CD31 (C), and anti S100a9 (D) antibodies. The alveolar and tubular epithelial system (B; Na-K-ATPase) is enveloped by the honeycomb-like vascular system (C; CD31). All neutrophils are visualized using anti-S100a9 staining (D). Scale bars; 50 µm.


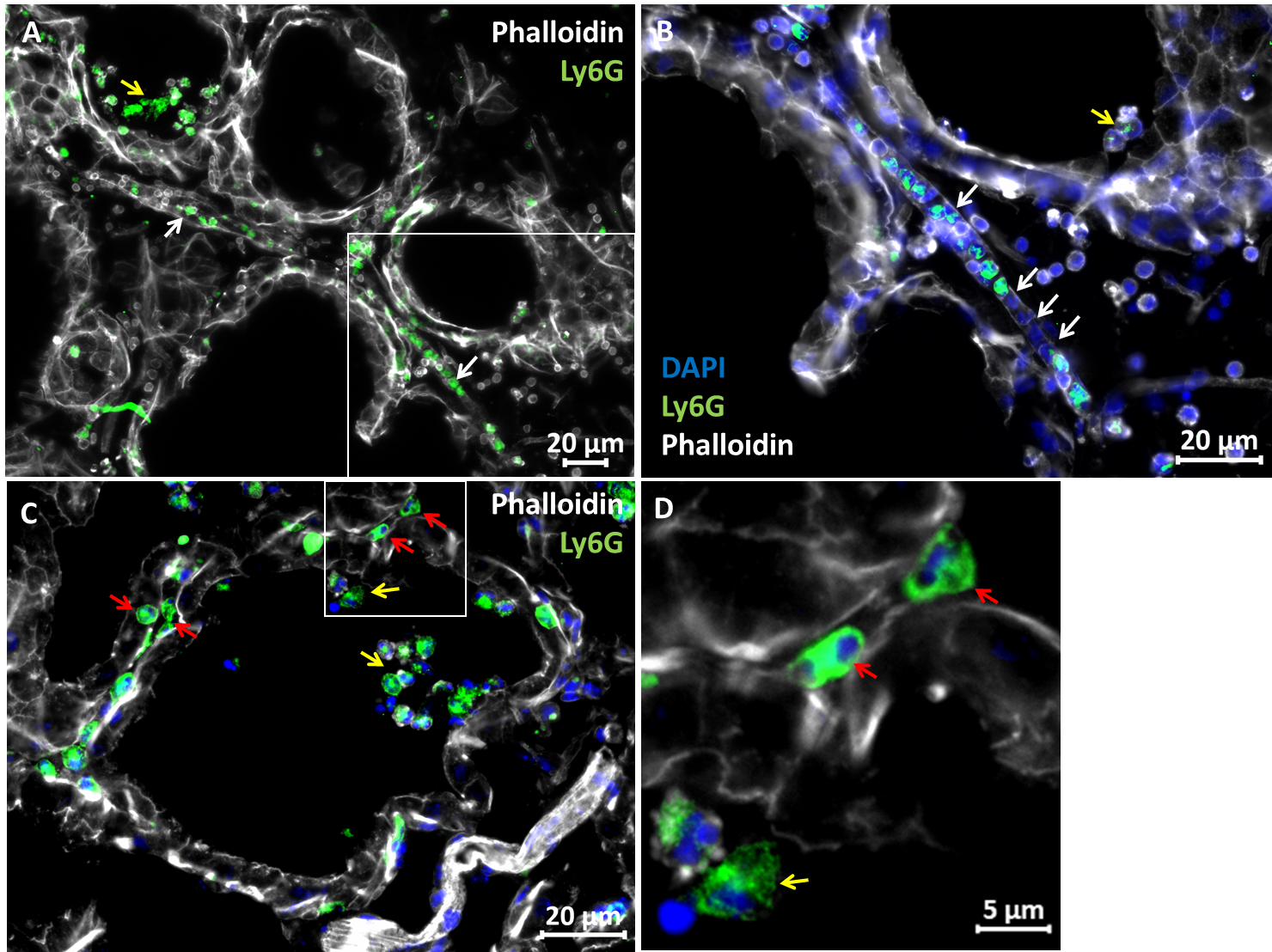


**Supplementary Figure 3. Trafficking of blood neutrophils into the alveolar milk space.** Mammary tissues from infected glands were stained using phalloidin (white in A-D), DAPI (blue in B-D), and Ly6G (green in A-D). Boxed areas in A and C are enlarged in B and D, respectively. Ly6G-positive neutrophils (green) are visible in capillaries and post capillaries venules (white arrows in A-B), and at various stages of their parenchymal and transepithelial journey (red arrows in C-D) into the alveolar milk space (yellow arrows in A-D). Scale bars; 20 µm (A-C) and 5 µm (D). Representative images from mammary glands 24 hours after IMM challenge with *E. coli* P4-NR bacteria.


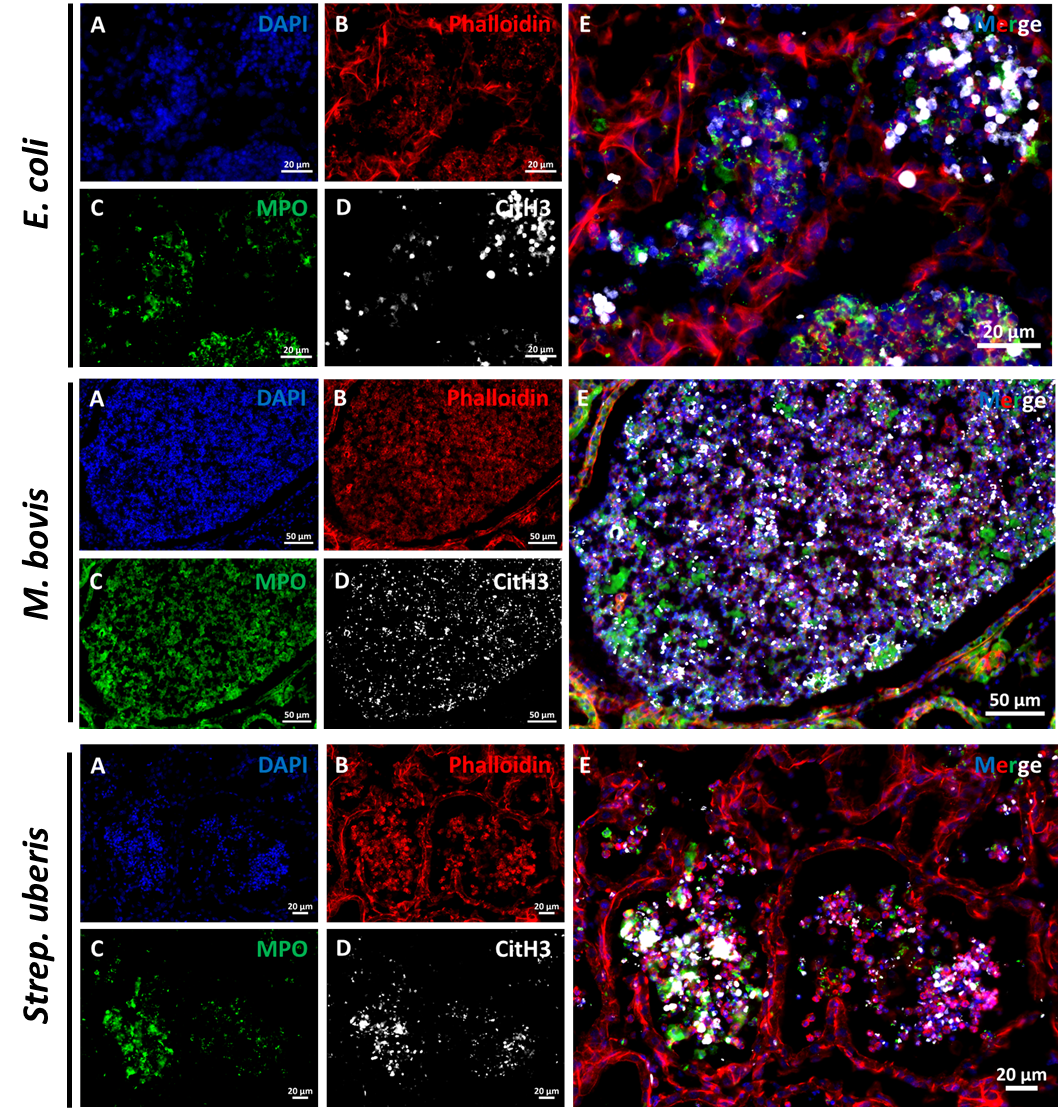


**Supplementary Figure 4.** Representative images of immunofluorescence staining for DAPI (blue), phalloidin (red), myeloperoxidase (green), and citrullinated histone H3 (white) in mouse mammary glands challenged with *E. coli* (top panel), *M. bovis* (middle panel) and Strep. uberis (bottom panel). Scale bars; 20µm (top and bottom panels) and 50µm (middle panel).


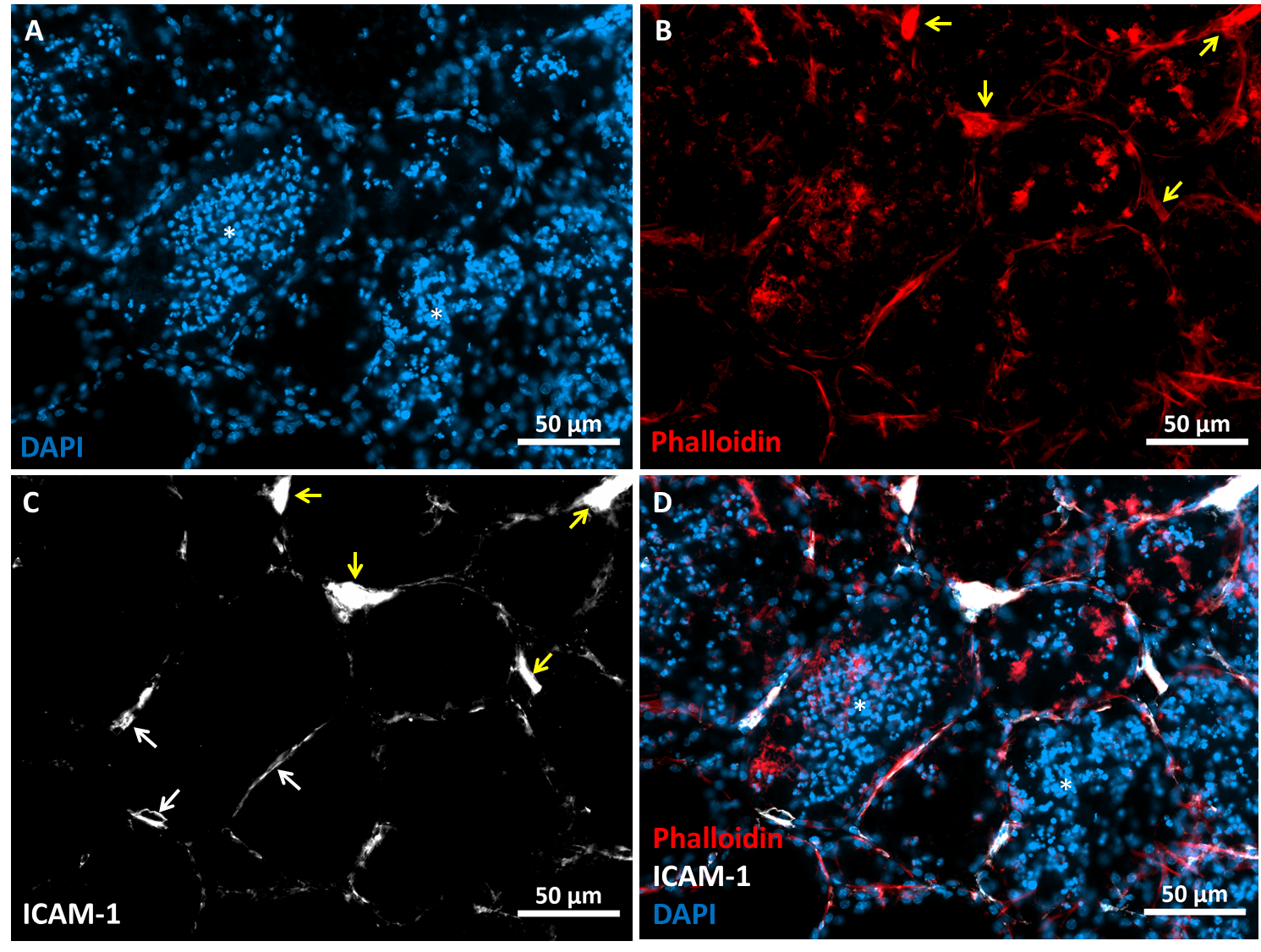


**Supplementary Figure 5. Expression of ICAM-1 in blood vessels of infected glands.** Mammary tissues from infected glands were stained using DAPI (blue in A and D), phalloidin (red in B and D), and anti-ICAM-1 antibodies (white in C and D). Numerous neutrophils are visible in the alveolar space (white asterisks in A and D). Leger blood vessels are intensely stained for phalloidin and ICAM-1 (yellow arrows in B and C). High expression of ICAM-1 in smaller perialveolar blood vessels in also visible (white arrows in C). Scale bars; 50 µm. Representative images from mammary glands 24 hours after IMM challenge with *E. coli* P4-NR bacteria.
